# In-Situ Grown Silver Nanoparticles on Nonwoven Fabrics Based on Mussel-Inspired Polydopamine for Highly Sensitive SERS Carbaryl Pesticides Detection

**DOI:** 10.3390/nano9030384

**Published:** 2019-03-06

**Authors:** Zhiliang Zhang, Tiantian Si, Jun Liu, Guowei Zhou

**Affiliations:** 1 Key Laboratory of Fine Chemicals in Universities of Shandong, School of Chemistry and Pharmaceutical Engineering, Qilu University of Technology (Shandong Academy of Sciences), Jinan 250353, China; sttsitiantian@163.com (T.S.); chgwzhou@126.com (G.Z.); 2 State Key Laboratory of Biobased Material and Green Papermaking, Qilu University of Technology (Shandong Academy of Sciences), Jinan 250353, China; 3 School of Light Industry Science and Engineering, Qilu University of Technology (Shandong Academy of Sciences), Jinan 250353, China

**Keywords:** polydopamine, in-situ reduction, non-woven fabrics, flexible surface-enhanced Raman scattering, carbaryl detection

## Abstract

The rapid sampling and efficient collection of target molecules from a real-world surface is fairly crucial for surface-enhanced Raman scattering (SERS) to detect trace pesticide residues in the environment and in agriculture fields. In this work, a versatile approach was exploited to fabricate a flexible SERS substrate for highly sensitive detection of carbaryl pesticides, using in-situ grown silver nanoparticles (AgNPs)on non-woven (NW) fabric surfaces based on mussel-inspired polydopamine (PDA) molecules. The obtained NW@PDA@AgNPs fabrics showed extremely sensitive and reproducible SERS signals toward crystal violet (CV) molecules, and the detection limit was as low as 1.0 × 10^−12^ M. More importantly, these NW@PDA@AgNPs fabrics could be directly utilized as flexible SERS substrates for the rapid extraction and detection of trace carbaryl pesticides from various fruit surfaces through a simple swabbing approach. It was identified that the detection limits of carbaryl residues from apple, orange, and banana surfaces were approximately decreased to 4.02 × 10^−12^, 6.04 × 10^−12^, and 5.03 × 10^−12^ g, respectively, demonstrating high sensitivity and superior reliability. These flexible substrates could not only drastically increase the collection efficiency from multifarious irregular-shaped matrices, but also greatly enhance analytical sensitivity and reliability for carbaryl pesticides. The fabricated flexible and multifunctional SERS substrates would have great potential to trace pesticide residue detection in the environment and bioscience fields.

## 1. Introduction

Carbaryl pesticides are extensively used in the agriculture, environment, and bioscience fields due to their broad generalizability and high insecticidal efficacy but low toxicity toward a variety of warm-blooded animals [1,2]. However, residual carbaryl pesticides could inhibit the activity of enzyme acetylcholinesterase, which is extremely essential for central nervous system function, and induce allergic hypersensitivity reactions. In severe cases, the enzyme inactivity could cause serious respiratory paralysis and deadly poisonous diseases for a lot of living organisms [3,4,5]. Due to above serious problems, the Codex Alimentarious Commission (CAC) have enacted a series of relative legislation to restrict the level of carbaryl pesticides released into the environment, and a number of technologies, such as gas chromatography–mass spectrometry (GC-MS), liquid chromatography–mass spectrometry (LC-MS), thin-Layer chromatography (TLC), and electrochemical sensors, have been exploited to detect the residues of carbaryl pesticides in the environment [2,3]. Although these well-established methods could be utilized to detect trace amounts of carbaryl pesticide residues, they are usually highly priced, time-consuming, labor-intensive, and often require complicated procedures of sample pretreatment [6,7]. In addition, the above methods require high amounts of toxic organic solvents and exhaustive cleanup treatments during the detection process of carbaryl pesticides [7,8,9].

The surface-enhanced Raman scattering (SERS) has high sensitivity and could provide rich molecular information, and has been an effective detection tool in environmental monitoring, food safety, and biotechnology science [10,11]. In the recent twenty years, considerable efforts have been devoted to improve the sensitivity, uniformity, and reliability of SERS substrates, and two typical strategies are adopted to effectively improve SERS signals [12]. In general, the common method is to control the morphology and components of SERS substrates by e-beam lithography, vapor deposition, and self-assembly techniques. The other one is to extract target molecules from a very complex surface to SERS substrates for highly sensitive trace detection in an effective and rapid method [13,14,15].

However, most of those common substrate materials are fairly rigid, such as glass sheets, silicon wafers, and porous alumina, and lack serious flexibility, making it difficult to extract the target analytes from the irregular-shaped matrices. This leads to low sample collection efficiency, thus limiting their application for in-situ analytes detection from irregular surfaces [16]. Recently, the emerging flexible SERS substrate materials have demonstrated an incredible potential to provide an in-situ sensitive SERS detection of target molecules from rugged or uneven surfaces [13,16]. For example, paper, cotton, sponge, and polymer materials are decorated with various metallic nanostructures and serve as SERS flexible substrates, which demonstrate good flexibility and efficiency for target molecule detection by the swabbing method. Despite the application prospects of flexible SERS substrates being fairly enormous, it still remains a big challenge to collect the targets from real-world surfaces and improve sampling efficiency [13,17]. At present, it is very urgent to exploit an approach to fabricate flexible and soft SERS substrates for rapid and efficient extraction of targets for highly sensitive SERS detection.

As flexible three-dimensional interconnected frameworks, non-woven (NW) fabrics possess good permeability, large specific surfaces, and high mechanical flexibility. These advantages promote the non-woven fabrics as promising candidates to fabricate flexible SERS substrates for surface contamination analysis [18,19]. Due to abundant amine and catechol groups in the molecule surfaces, dopamine (DA) molecules exhibit similar molecular structures and properties with mussels, such as superior self-polymerization, in-situ reduction, and special recognition capability [20,21]. In particular, the DA self-polymerization provides an efficient and simple approach to surface modification. Moreover, polydopamine (PDA) possesses strong complexation with metallic cations and reduces them spontaneously in-situ into metallic nanostructures by oxidizing catechols into the corresponding quinine groups [22,23,24,25]. As a result, PDA could serve as a versatile molecule platform to generate uniform silver nanoparticles (AgNPs) with desirable morphology and coverage density on NW fabrics, which provides a promising opportunity to fabricate a flexible SERS substrate for trace carbaryl pesticide detection [26,27,28].

Inspired by these distinctive advantages of PDA molecules, in this work, a simple and versatile approach was exploited to fabricate flexible SERS substrates for highly sensitive carbaryl detection by AgNPs in-situ grown onto the NW fabric surfaces. By regulating the reaction time between catechol groups and [Ag(NH_3_)_2_]^+^ ions, a controllable density of AgNPs was in-situ grown onto the surfaces of NW fabrics. These NW@PDA@AgNPs fabrics exhibited high sensitivity and superior reproducibility, and the detection concentration limit was as low as to 1.0 × 10^−12^ M toward crystal violet (CV) molecules. More importantly, these NW@PDA@AgNPs fabrics could be directly utilized for conformal SERS detection of carbaryl pesticides from complex surfaces through a simple swabbing approach, and achieved a high sensitivity and superior reliability. It was identified that the detection limit of carbaryl residues from apples, oranges, and bananas approximately decreased to 4.02 × 10^−12^, 6.04 × 10^−12^, and 5.03 × 10^−12^ g, respectively. These NW@PDA@AgNPs fabrics could not only drastically increase the sample collection efficiency, but also be used as flexible SERS substrates for real sample detection with negligible pretreatment. The fabrication approach would demonstrate enormous potential to construct versatile SERS substrates for highly sensitive and reliable detection of traces of carbaryl pesticide residues in the agriculture, environment, and bioscience fields.

## 2. Materials and Methods

### 2.1. Chemicals and Materials

Silver nitrate, dopamine, tris-base (≥99.9%), crystal violet (CV), and carbaryl were obtained from Sigma–Aldrich (St. Louis, MO, USA). Ethanol and acetone wereobtained from Beijing Chemical Co. (Beijing, China). The pristine NWfabrics were mainly composed of hemicellulose, cellulose, and lignin, and the major elements were C, H, and O, which werepurchased from Asahi Kasei Corporation Co., Ltd. (Tokyo, Japan). The other chemicals were of analytical or high-reagent grades.

### 2.2. Decoration of NW Fabrics with PDA Molecules

The NW fabrics were cut into small pieces (0.8 × 0.8 cm^2^) and ultrasonically cleaned in ethanol, acetone, and deionized (DI) water. Then, the cleaned NW fabrics were immersed in the tris-HCl buffer solution, and the concentration of tris-HCl buffer was controlled at 50 mM. The DA (concentration of about 2 mg L^−1^) was slowly added into the above solution and reacted for 24 h at pH = 8.5 under ultrasonic agitation. Finally, the excess PDA on NW fabric surfaces was washed adequately with DI water and ethanol, and dried under nitrogen atmosphere for further SERS analysis. The obtained composites were denoted as NW@PDA fabrics.

### 2.3. In-situ Grown AgNPs on the NW@PDA Surface

To achieve in-situ growth of AgNPs on the NW@PDA surface, the obtained NW@PDA fabrics were fully immersed into the fresh [Ag(NH_3_)_2_]^+^ solution with stirring. The fresh [Ag(NH_3_)_2_]^+^ solution was prepared by adding excessive NH_3_·H_2_O into 6 mg L^−1^ AgNO_3_ solution with stirring until the precipitates completely subsided and a clear solution was formed. By regulating the immersion time, a controllable density and coverage of AgNPs were in-situ grown on the surface of NW@PDA fabrics. After that, the obtained composites were washed several times with DI water, and the obtained composites were denoted as NW@PDA@AgNPs fabrics.

### 2.4. Detection of CV on NW@PDA@AgNPs Fabrics

To evaluate the SERS sensitivity and reproducibility of NW@PDA@AgNPs fabrics, CV was used as the Raman model probe, and the responding SERS spectra were collected. In brief, 50 μL of CV solution in the range of 10^−5^–10^−12^ M was prepared and dropped on the surface of NW@PDA@AgNPs fabrics. The NW@PDA@AgNPs fabrics were dried under nitrogen atmosphere for Raman signals detection.

### 2.5. Detection of Carbaryl Residues on the Flexible NW@PDA@AgNPs Fabrics

To simulate the real-world SERS application, all fruits used in the experiments, such as apples, oranges, and bananas, were washed with ultrapure water three times. Subsequently, 25 μL of carbaryl pesticide solution in the range of 10^−3^–10^−10^ M was sprayed onto the surfaces of above apples, oranges, and bananas, and the spray areas were controlled in 1 × 1 cm squares. Then, the above fruits were carefully swabbed with the flexible NW@PDA@AgNPs fabrics, which were wet by ethanol in advance. After the solvent evaporation, SERS detection was conducted on the NW@PDA@AgNPs fabrics.

### 2.6. Characterization

The X-ray diffraction (XRD) patterns were collected on a D8 Advance X-ray diffractometer (Bruker, Germany). The microscopic morphologies and energy-dispersive spectroscopies of the above samples were taken on a S-8220 scanning electron microscope (Hitachi, Japan). Thermo Scientific ESCALabXi^+^ was used to collect X-ray photoelectron spectroscopy (XPS) with 200 W monochromatic Al Kα radiation (Thermo Fisher, Waltham, MA, USA). Raman analyses were performed on a inVia9 Raman Microscope using a 532 nm laser irradiation (Renishaw, UK), with a 50× objective focusing the laser beam onto a spot with approximately 1um diameter. The intensity of irradiation laser was controlled at 1 mW to activate the samples during the whole process, and all SERS signals were gathered for about 1 s.

## 3. Results and Discussion

**Figure 1:**
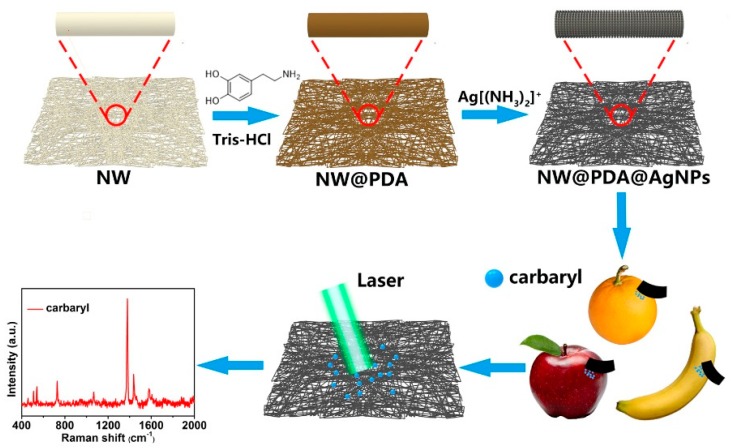
Schematic of the fabrication of non-woven polydopamine/silver nanoparticle (NW@PDA@AgNPs) fabrics for highly sensitive detection of carbaryl pesticide residues.

Figure 1 illustrates our strategy to construct flexible SERS substrates for highly sensitive detection of carbaryl pesticide residues by AgNPs in-situ grown onto the surfaces of non-woven fabrics with mussel-inspired PDA molecules. Briefly, the loose and precleaned masses of NW fabrics were immersed into a DA solution, and PDA layers with abundant amino and catechol groups were spontaneously formed on the NW surface via DA self-polymerization. Due to the superior complexation, the amino and catechol groups on PDA layers were complexed with silver ions after dipping into the [Ag(NH_3_)_2_]^+^ solution. Subsequently, the NW@PDA@AgNPs fabrics with controllable morphology were fabricated via in-situ reduction of [Ag(NH_3_)_2_]^+^ cations into AgNPs by oxidizing catechols into the corresponding quinine groups. As the NW@PDA@AgNPs fabrics were directly used as flexible SERS substrates to collect trace carbaryl pesticides from various fruit surfaces through a swabbing approach, a high SERS detection sensitivity and reliability for trace carbaryl pesticides could be achieved due to their excellent flexibility and collection efficiency.

**Figure 2:**
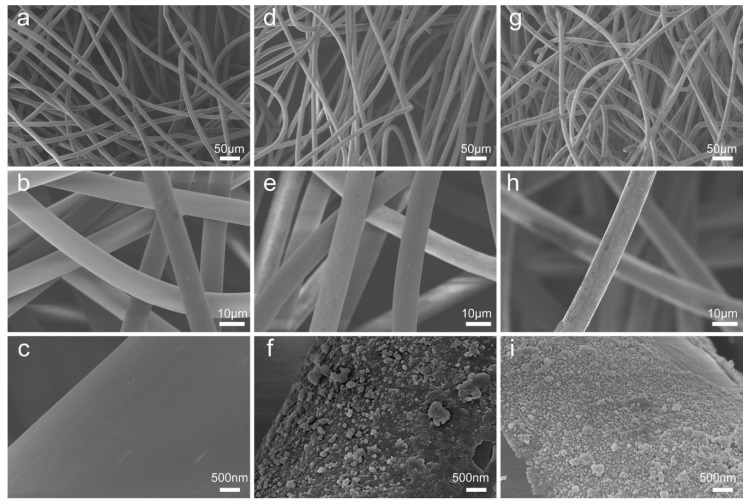
SEM images of the pristine non-woven (NW) fabrics (**a**–**c**), non-woven polydopamine (NW@PDA) fabrics (**d**–**f**), and NW@PDA@AgNPs fabrics (**g**–**i**).

To demonstrate the changes in surface morphology and element composition after respective DA modification, the pristine NW, NW@PDA, and NW@PDA@AgNPs fabrics were characterized by SEM. From Figure 2a–c, the fibers of pristine NW fabrics were very smooth with no nanostructures on the surfaces. After immersion in the DA solution, however, the NW fabrics became dark and rough due to the functional PDA layer formation via spontaneous DA self-polymerization (Figure 2d–f, Figure S1a). After further treatment with [Ag(NH_3_)_2_]^+^ solution, silver ions were in-situ reduced into AgNPs, and each of fiber in the NW fabrics was tightly covered with AgNPs (Figure 2g–i), and the sizes of the AgNPs were controlled in the range of 30–60 nm, with spherical morphologies. When comparing the energy dispersive spectroscopy (EDS) results with those of the pristine NW and NW@PDA fabrics (Figure S1b–d), the silver peaks emerged and showed stronger intensities for the NW@PDA@AgNPs fabrics, which was ascribed to having plenty of AgNPs on the surfaces of the NW@PDA@AgNPs fabrics. Furthermore, the interfacial interactions between AgNPs and NW fabrics were fairly strong, which provided a flexible SERS substrate to collect carbaryl pesticides from complex surfaces through a simple swabbing approach for SERS detection [28].

**Figure 3:**
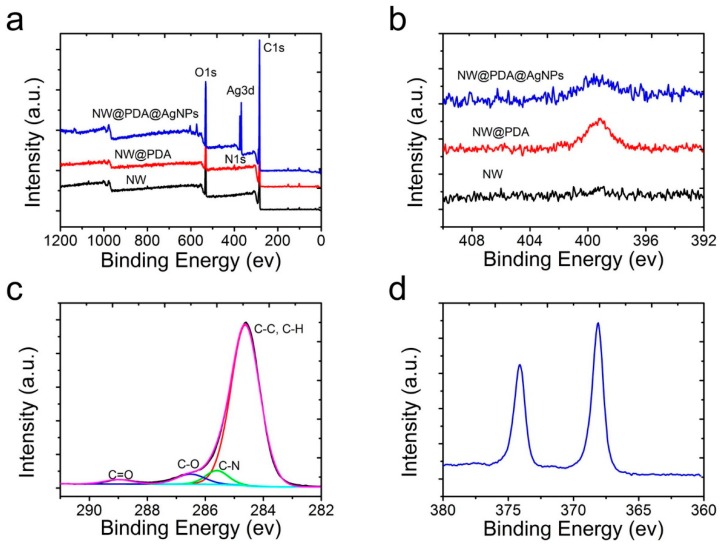
(**a**) X-ray photoelectron spectroscopy (XPS) survey of the pristine NW, NW@PDA, and NW@PDA@AgNPs fabrics. (**b**) The changes of N1s spectra in the pristine NW, NW@PDA, and NW@PDA@AgNPs fabrics. (**c**) Narrow-scan XPS spectra of C1s in NW@PDA@AgNPs fabrics. (**d**) High-resolution spectrum of Ag3d in NW@PDA@AgNPs fabrics.

In order to further verify the element changes after mussel-inspired PDA modification, XPS was used to characterize the pristine NW, NW@PDA, and NW@PDA@AgNPs fabrics. From the XPS survey spectra in Figure 3a, the C, N, and O elements at 284.80, 399.91, and 532.35 eV were all clearly detected, which were attributed to the C 1s, N 1s, and O 1s, respectively. Compared to the XPS results in pristine NW and NW@PDA fabrics, the characteristic emission peaks of Ag3d at 368.14 eV clearly appeared in the XPS spectrum of NW@PDA@AgNPs fabrics, demonstrating that AgNPs were successfully deposited on NW@PDA fabrics via in-situ reduction. From Figure 3b, the N element in the NW@PDA increased due to the formation of DA self-polymerization. However, it showed a sharp decrease as the AgNPs were in-situ formed on the surfaces of NW@PDA@AgNPs fabrics. Moreover, the high-resolution C1s spectra were shown in Figure 3c for the NW@PDA@AgNPs fabrics. From the fitting results, four peaks were displayed at 284.55, 285.80, 286.75, and 288.95 eV, which were assigned to the C–C, C–N, C–O, and C=O groups, respectively. As shown in Figure 3d, the binding energies for Ag 3d5/2 and Ag 3d3/2 were identified at 368.12 and 374.16 eV, respectively. The very narrow peak widths indicated that only a single zero valence element, silver, existed in the NW@PDA@AgNPs fabrics.

From the XRD spectra in Figure S2, the pristine NW and NW@PDA fabrics presented three obvious peaks at 17.26°, 22.92°, and 25.36°, respectively, originating from the crystalline structures of hemicellulose, cellulose, and lignin. The typical diffraction peaks of NW fabrics demonstrated no obvious changes after PDA modification, which suggested that the crystal structures of NW fabrics remained unchanged [19]. Furthermore, the representative diffraction peaks of the silver element appeared in the NW@PDA@AgNPs sample at 37.96°, 44.18°, 64.42°, and 77.28°, which corresponded with 111, 200, 220, and 311 crystal planes of face-centered cubic silver crystal (JCPDS No.83-0718). According to all the above results, the AgNPs were successfully in-situ grown on the NW fabric surfaces based on the mussel-inspired PDA modification, and the NW@PDA@AgNPs fabrics would be excellent flexible SERS substrates to detect trace carbaryl pesticides.

**Figure 4:**
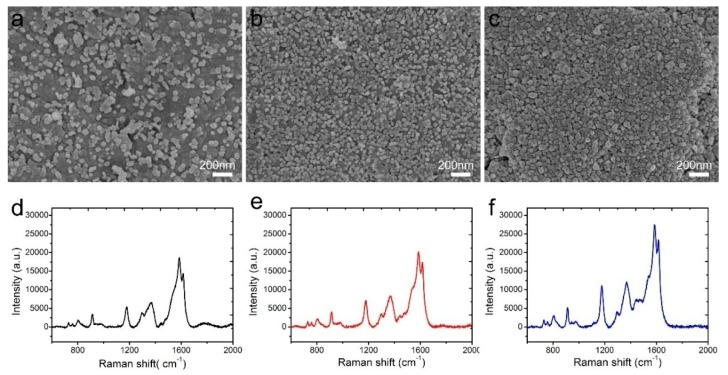
SEM images of NW@PDA fabrics after immersing in [Ag(NH_3_)_2_]^+^ solution for (**a**) 4, (**b**) 8, and (**c**) 12 h. (**d**–**f**) Surface-enhanced Raman scattering (SERS) spectra of crystal violet (CV) collected on the 4, 8, and 12 h NW@PDA@AgNPs fabrics.

The AgNP morphologies of NW@PDA@AgNPs fabrics were extremely crucial to obtain highly sensitive and reliable SERS detection of trace carbaryl pesticides. In order to obtain the highest SERS signal intensities, the AgNP morphologies were regulated by controlling the immersion time of NW@PDA fabrics in the [Ag(NH3)_2_]^+^ solution. As shown in Figure 4a, AgNPs were gradually formed and randomly distributed on the NW@PDA@AgNPs fabrics at the initial time, and the sizes of the AgNPs were in the range of 30–60 nm. With the increase of immersion time, the amount of AgNPs gradually grew larger on the NW@PDA@AgNPs fabrics with more [Ag(NH_3_)_2_]^+^ cations turning into AgNPs through the in-situ reduction, and consequently, more nanogaps were formed among the AgNPs (Figure 4b). As the immersion time further increased to 12 h, the AgNPs tended to pack together, resulting more interstices and nanogaps between adjacent AgNPs.

CV with a concentration of 1 × 10^−5^ M was used as a probe molecule to evaluate the performance of these NW@PDA@AgNPs fabrics as flexible SERS substrates. From Figure 4d–f, the Raman enhancement effect increased with the immersion time, and it achieved maximum enhancement when the immersion time reached to 12 h. These results were ascribed to abundant SERS “hot spot” formation on the NW@PDA@AgNPs fabrics, which induced a strong electromagnetic enhancement.

**Figure 5:**
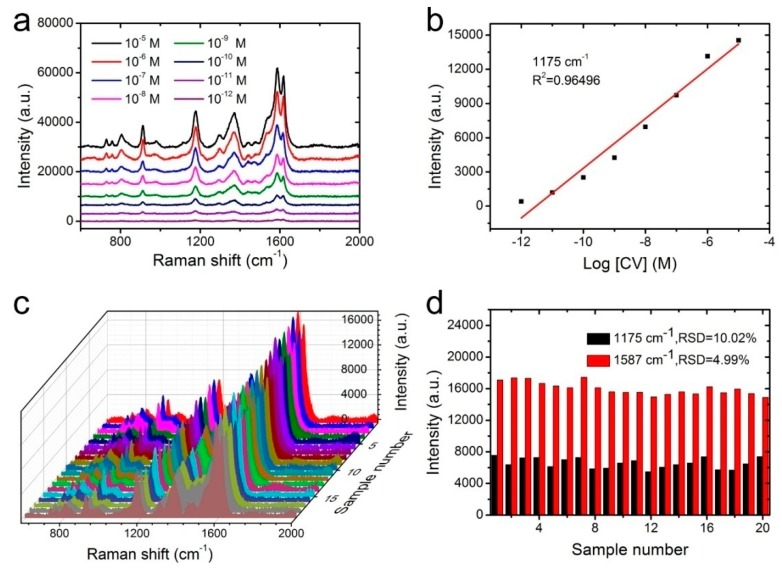
(**a**) SERS spectra of CV at different concentrations on the NW@PDA@AgNPs fabrics. (**b**) The calibration curve of characteristic peak intensity at 1175 cm^−1^ with the logarithm values of sample concentration. (**c**) The stability of SERS substrate from 20 random points probed with CV molecules. (**d**) Intensity distribution of CV at 1175 and 1587 cm^−1^, collected from 20 different NW@PDA@AgNPs fabrics.

The high sensitivity was fairly important for SERS substrates to realize the trace component and even single-molecule detection. In order to verify the sensitivity of NW@PDA@AgNPs fabrics as flexible SERS substrates, CV molecules with concentrations of 1 × 10^−5^ to 1 × 10^−12^ M were used to estimate the Raman enhancement effect. As shown in Figure 5a, the intensity of CV SERS signals gradually decreased as the concentration decreased from 1 × 10^−5^ to 1 × 10^−12^ M. Even if the CV concentration was lowered to 1 × 10^−12^ M, the typical bands at 912, 1175, 1587, and 1618 cm^−1^ still obviously appeared. This indicated that the flexible NW@PDA@AgNPs fabrics had a high SERS sensitivity, which was attributed to the large number of “hot spots” for SERS detection on the NW@PDA@AgNPs fabrics. Moreover, Figure 5b showed that the SERS intensity demonstrated a good linear relationship to CV concentration with a correlation coefficient R^2^ = 0.9649, which was very favorable to achieve the quantitative determination for the target molecules from various surfaces [29,30]. According to the previous calculation method in the supporting information, the enhancement factor (EF) could be calculated as 7.02 × 10^6^ (Figure S3a).

Besides high sensitivity, the signal reproducibility was also a crucial factor for the SERS substrate to obtain reliable detection results. In order to verify the reproducibility, the Raman signals of CV (1 × 10^−7^ M) were recorded from 20 randomly selected spots on the NW@PDA@AgNPs fabrics. As shown in Figure 5c, all the selected spots revealed relatively consistent Raman signals at 912, 1175, 1373, and 1587 cm^−1^, and satisfied the requirements of uniform reinforced substrates. Moreover, the relative standard deviations (RSD) of the Raman vibration at 1175 and 1587 cm^−1^ were approximately 10.02% and 4.99% (≤20%), respectively (Figure 5d) [31]. These statistical results proved that the NW@PDA@AgNPs fabrics fabricated by our strategy could serve as flexible SERS platforms to achieve excellent sensitivity and superior reproducibility.

**Figure 6:**
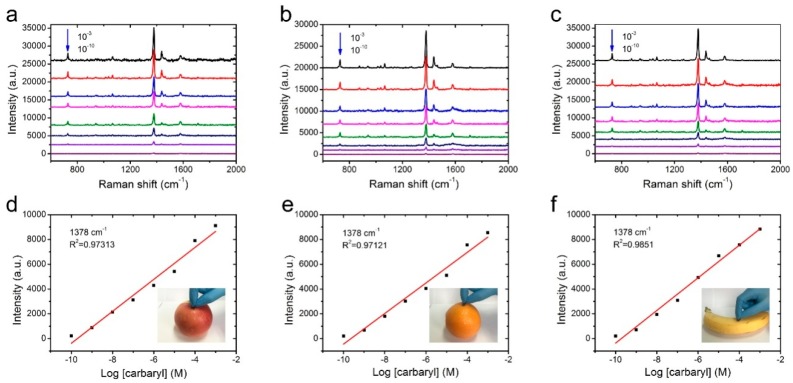
SERS spectra of the NW@PDA@AgNPs fabrics through swabbing extraction with different carbaryl concentrations on (**a**) apple, (**b**) orange, and (**c**) banana surfaces. The respective calibration curves for carbaryl on (**d**) apple, (**e**) orange, and (**f**) banana surfaces through swabbing extraction.

Swabbing the surfaces with a versatile and flexible SERS substrate is a highly efficient and practical method that could greatly enhance the sample collection from a real-world surface. To confirm the applicability of NW@PDA@AgNPs fabrics for collecting the carbaryl residues from the surfaces of various fruits, the carbaryl pesticide was dissolved in alcohol and diluted to different concentrations in the range of 1 × 10^−3^ to 1 × 10^−10^ M. The diluted carbaryl pesticides were sequentially sprayed on the surfaces of apples, oranges, and bananas, and then these surfaces were dried at room temperature. The fabricated NW@PDA@AgNPs fabrics were used as flexible substrates to collect the residues of carbaryl pesticides by the surface swabbing method, and the residues were detected on a Renishaw inVia9 Raman Microscope with a 532 nm laser irradiation. From Figure 6a–c, the representative peaks located at 729, 1378, 1436, and 1577 cm^−1^ obviously appeared, and were well in accordance with the related reports for carbaryl pesticide SERS detection [19].

As the concentration decreased from 1 × 10^−3^ to 1 × 10^−10^ M, the peak intensity of carbaryl pesticide demonstrated a sharp decline. As the concentration was lowered to 1 × 10^−10^ M, the typical absorption bands of carbaryl at 1378 and 1577 cm^−1^ could be still distinctly observed, indicating that the NW@PDA@AgNPs fabrics had high SERS sensitivity to carbaryl pesticide detection. In order to further confirm the enhancement effect, the normal Raman spectra of carbaryl on silicon wafers were detected and used as the benchmark data. According to the previous calculation method [30], the EFs could be calculated to be 1.26 × 10^6^ (apple), 1.19 × 10^6^ (orange), and 1.36 × 10^6^ (banana) (Figure S3b–d), which were higher than those of the related methods [32,33]. In fact, due to the strong electromagnetic coupling, the AgNPs on the NW@PDA@AgNPs fabrics could produce large Raman signal enhancements [25], and a lot of SERS “hot spots” were spontaneously formed from the nanogaps and interstices in NW@PDA@AgNPs fabrics, which was fairly beneficial to achieve the high SERS sensitivity.

Moreover, the peak intensity of carbaryl pesticide demonstrated a good linear relationship with the corresponding logarithmic concentration, and the correlation coefficients for carbaryl pesticide on the apple, orange, and banana surfaces were 0.9731, 0.9712, and 0.9851, respectively. In addition, when calculated by the concentration and volume, there was only 4.02 × 10^−12^ g of carbaryl pesticide on the apple surface collected. With the same quantitative method, the carbaryl pesticides on the orange and banana surfaces were collected, and these could still be detected even at amounts of 6.04 × 10^−12^ and 5.03 × 10^−12^ g, respectively, by the direct swabbing method. These analysis results fully proved that the NW@PDA@AgNPs fabrics could serve as flexible SERS substrates to efficiently collect the carbaryl pesticides and provide satisfactory quantitative analysis results from a real-world surface [34,35,36].

**Figure 7:**
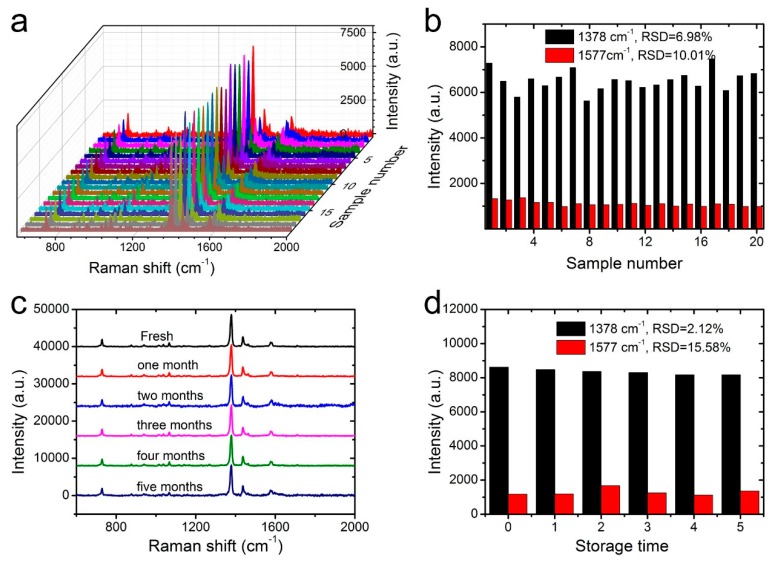
(**a**) The SERS homogeneity of carbaryl on the NW@PDA@AgNPs fabrics collected from 20 different points. (**b**) The corresponding intensity variations at 1378 and 1577 cm^−1^ in the histogram. (**c**) The Raman spectra of carbaryl on the fresh NW@PDA@AgNPs fabrics and those with storage time of 1 or more months. (**d**) The corresponding intensity variations at 1378 and 1577 cm^−1^ in the histogram.

Apart from the high SERS sensitivity, the homogeneity and stability were two other crucial factors for the NW@PDA@AgNPs fabrics to achieve the reliable SERS detection as flexible SERS substrates. To evaluate the SERS homogeneity, 20 points were randomly selected from the surfaces of NW@PDA@AgNPs fabrics, and the responding Raman signals of carbaryl pesticide were collected. As shown in Figure 7a, the intensities of the typical band at 1378 and 1577 cm^−1^ exhibited almost identical absorption peaks. The RSD values of the Raman peaks at 1378 and 1577 cm^−1^ were approximately 6.98% and 10.01%, respectively (Figure 7b), which fully proved that the NW@PDA@AgNPs fabrics could obtain highly uniform and homogeneous SERS signals.

Moreover, the Raman signal stability of NW@PDA@AgNPs fabrics was investigated in detail and demonstrated in Figure 7c,d. From Figure 7c, which compares the fresh sample with those even stored for more than five months, the Raman signal intensities of SERS peaks at 1378 and 1577 cm^−1^ demonstrated no significant decrease, and the calculated RSDs were approximately 2.12% and 15.58% (Figure 7d), indicating an excellent stability of NW@PDA@AgNPs fabrics for practical SERS detection. From all the above analyses, the NW@PDA@AgNPs fabrics could simultaneously acquire superior sensitivity, homogeneity, and stability, and would have great potential applications to achieve the detection of trace pesticide residues from a real-world surface.

## 4. Conclusions

In summary, an efficient strategy was explored to construct a flexible SERS substrate for highly sensitive and reliable carbaryl pesticide detection by AgNPs in-situ grown onto the NW fabric surfaces based on mussel-inspired DA molecules. The NW@PDA@AgNPs fabrics exhibited an extremely sensitive and reproducible SERS response to CV molecules, and the detection limit was as low as 1.0 × 10^−12^ M. More importantly, these NW@PDA@AgNPs fabrics could serve as flexible SERS substrates to extract and detect trace carbaryl pesticides from various fruits by a simple swabbing approach. In addition, the NW@PDA@AgNPs fabrics demonstrated rapid detection of trace carbaryl residues with high collection efficiency, and the detection limits from apple, orange, and banana surfaces were approximately down to 4.02 × 10^−12^, 6.04 × 10^−12^, and 5.03 × 10^−12^ g, respectively. These NW@PDA@AgNPs fabrics could be used as versatile SERS platforms for trace carbaryl residues in the environment, agriculture, and bioscience fields.

## Supplementary Materials

The following are available online at https://www.mdpi.com/2079-4991/9/3/384/s1, Figure S1: (a) Optical images of the pristine NW, NW@PDA and NW@PDA@AgNPs fabrics. The responding EDS spectra of (b) the pristine NW fabrics, (c) the NW@PDA fabrics and (d) the NW@PDA@AgNPs fabrics respectively. Figure S2: XRD patterns of the pristine NW, NW@PDA and NW@PDA@AgNPs fabrics. Figure S3: (a) SERS spectra of 1 × 10^−9^ M CV on the NW@PDA@AgNPs fabrics (black) and Raman spectrum of 1 × 10^−3^ M CV on silicon wafer substrate (red). SERS spectra (black) of carbaryl on the NW@PDA@AgNPs fabrics collected from (b) apple, (c) orange, and (d) banana surface, and the Raman spectra (red) of carbaryl on silicon wafer substrate.
