# Supplementary material for: In-Situ Grown Silver Nanoparticles on Nonwoven Fabrics Based on Mussel-Inspired Polydopamine for Highly Sensitive SERS Carbaryl Pesticides Detection
Source: Nanomaterials (Basel). 2019 Mar 6;9(3):384. doi: 10.3390/nano9030384 (PMC6473996; doi:10.3390/nano9030384)
Supplement: Supplementary file 1 [file nanomaterials-09-00384-s001.pdf]

Supporting Information

In-situ Grown Silver Nanoparticles toward Nonwoven Fabrics Based on Mussel-inspired Polydopamine for Highly Sensitive SERS Carbaryl Pesticides Detection

Zhiliang Zhang [1,2,*], Tiantian Si [1], Jun Liu [3,*] and Guowei Zhou [1]

[1] Key Laboratory of Fine Chemicals in Universities of Shandong, School of Chemistry and Pharmaceutical Engineering, Qilu University of Technology (Shandong Academy of Sciences), Jinan, 250353, China

[2] State Key Laboratory of Biobased Material and Green Papermaking, Qilu University of Technology (Shandong Academy of Sciences), Jinan, 250353, China

[3] School of Light Industry Science and Engineering, Qilu University of Technology (Shandong Academy of Sciences), Jinan, 250353, China

* Correspondence: zhzhl@iccas.ac.cn, liujun6621@163.com; Tel.: +86-053189631632

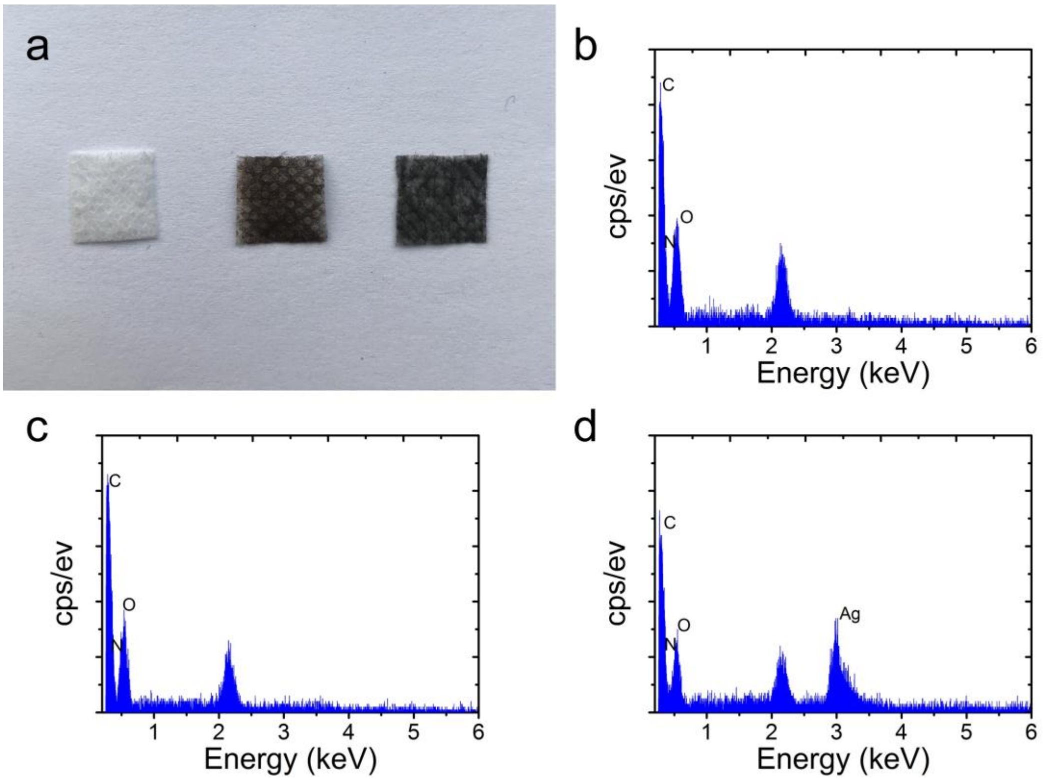

Figure 1. (**a**) Optical images of the pristine NW, NW@PDA and NW@PDA@AgNPs fabrics. The responding EDS spectra of (**b**) the pristine NW fabrics, (**c**) the NW@PDA fabrics and (**d**) the NW@PDA@AgNPs fabrics respectively.

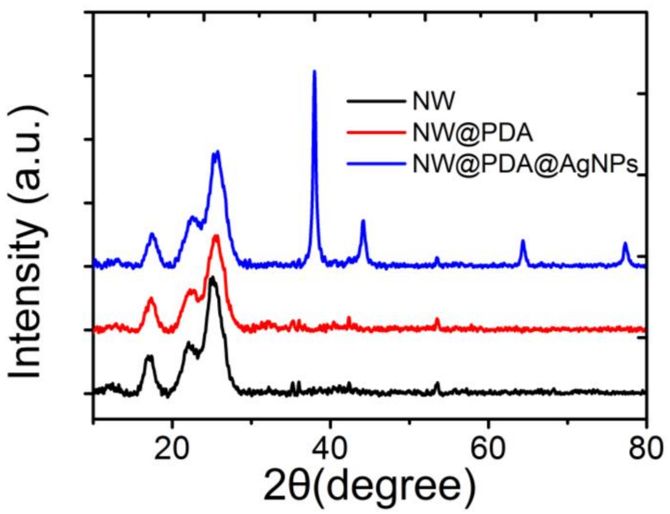

Figure 2. XRD patterns of the pristine NW, NW@PDA and NW@PDA@AgNPs fabrics**.**

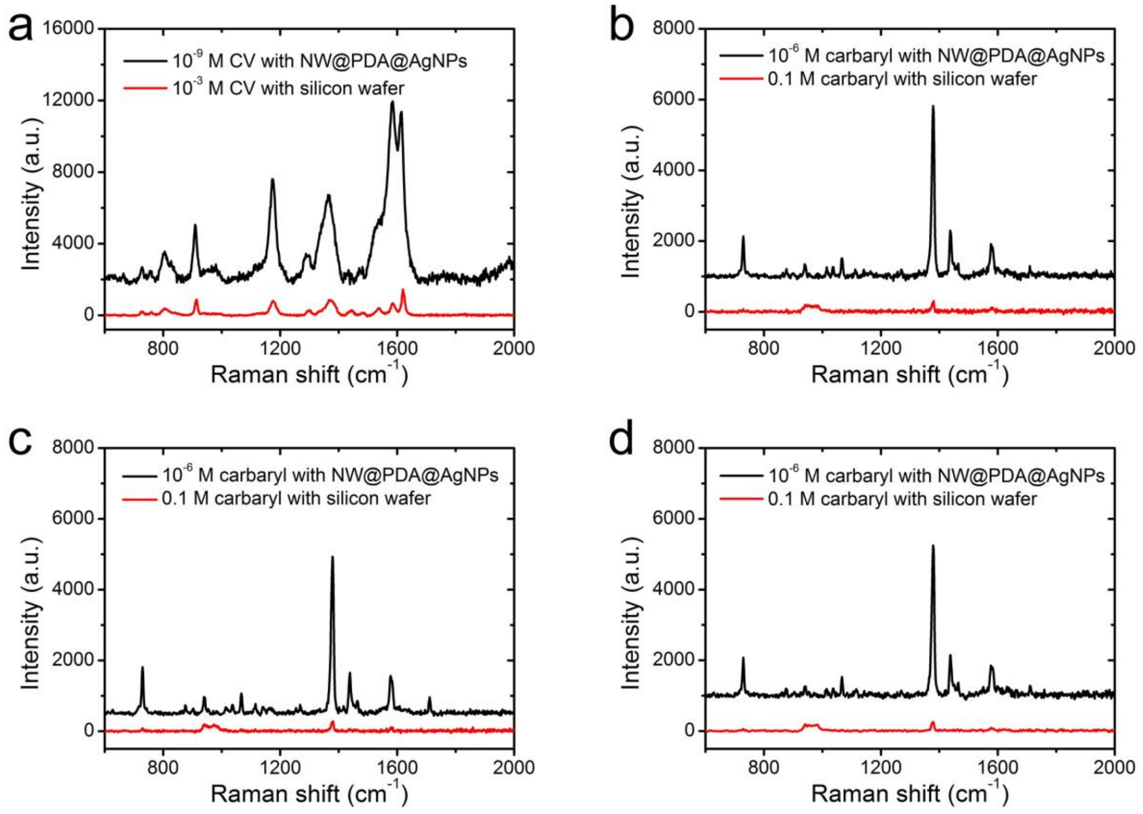

Figure 3. (**a**) SERS spectra of 1 × 10^{-9} M CV on the NW@PDA@AgNPs fabrics (black) and Raman spectrum of 1 × 10^{-3} M CV on silicon wafer substrate (red). SERS spectra (black) of carbaryl on the NW@PDA@AgNPs fabrics collected from (**b**) apple, (**c**) orange, and (**d**) banana surface, and the Raman spectra (red) of carbaryl on silicon wafer substrate.

Calculation of Enhancement Factor

The EF was determined by computing the ratio of SERS to normal Raman scattering (RS) of probe molecules, supposing that all the measured molecules within the laser spot are illuminated and contribute to the SERS spectra and the Raman spectra. EF can be calculated by the following expression [1]:

$$EF = \frac{I_{SERS} / N_{\mathrm{SERS}}}{I_{RS} / N_{\mathrm{RS}}} \tag{1}$$

where I_{SERS} and I_{RS} correspond to the SERS and RS intensities, respectively, and N_{SERS} and N_{RS} represented the number of molecules on the detection substrates within the laser spot.

The measurements were conducted under identical experimental conditions (laser wavelength, laser power, microscope objective/lenses, spectrometer, etc.). For SERS experiment, a certain concentration C_{SERS} and volume V_{SERS} CV ethanol solution was dispersed to an area S_{SERS} on the NW@PDA@AgNPs fabrics. For normal Raman experiment, dispersed a certain volume V_{RS} and concentration C_{RS} CV ethanol solution to an area of S_{RS} on cleaned silicon wafer and dried in the air. The above equation expression could become as followed:

$$EF = \frac{I_{SERS}}{I_{RS}} \cdot \frac{S_{SERS} V_{RS} C_{RS}}{S_{RS} V_{SERS} C_{SERS}} \tag{2}$$

For normal Raman experiment, 40 μL of 10^{-3} M CV ethanol solution was dispersed on a clean silicon wafer, and dried to form CV solid thin film. For SERS experiment, 50 μL of 10^{-9} M CV ethanol solution was dispersed to an area of 0.8 × 0.8 cm^2 for NW@PDA@AgNPs substrate. The SERS spectrum and the Raman spectrum of CV were shown in Figure S3a. The intensities at 1175 cm^{-1} were 803 units on silicon wafer and 5637 units for NW@PDA@AgNPs fabrics. According to the above equation, the average EF was calculated to be 7.02 × 10^6 for the NW@PDA@AgNPs fabrics.

For detecting of carbaryl residues, 25 μL of 10^{-6} M carbaryl ethanol solution sprayed onto the surface of apples, oranges and bananas respectively and swabbed with the NW@PDA@AgNPs fabrics (0.8 × 0.8 cm^2) to obtain the SERS spectra. In order to obtain Raman spectra, 20 μL of 0.1 M carbaryl ethanol solution was sprayed onto silicon wafer. The SERS intensities of wiping on the surface for three fruits were 4821, 4248, 4429 units, and Raman intensities were 304, 283, 259 units respectively. Figure S3 shown the Raman spectra and the SERS spectra of carbaryl from the as-prepared substrates. So the average EF for apples, oranges and bananas were calculated to 1.26 × 10^6, 1.19 × 10^6 and 1.36 × 10^6, respectively.

References

1. Huang, Z.; Meng, G.; Huang, Q.; Yang, Y.; Zhu, C.; Tang, C. Improved SERS Performance from Au Nanopillar Arrays by Abridging the Pillar Tip Spacing by Ag Sputtering. *Adv. Mater.* **2010**, *22*, 4136–4139.
